# Comparison of Three Glomerular Filtration Rate Estimating Equations with 24-Hour Urine Creatinine Clearance Measurement in Potential Living Kidney Donors

**DOI:** 10.1155/2023/2022641

**Published:** 2023-06-15

**Authors:** Fernando Giron-Luque, Andrea Garcia-Lopez, Yenny Baez-Suarez, Nasly Patino-Jaramillo

**Affiliations:** ^1^Department of Transplant Surgery, Colombiana de Trasplantes, Bogotá, Colombia; ^2^Department of Transplant Research, Colombiana de Trasplantes, Bogotá, Colombia

## Abstract

**Background:**

The accuracy of the measurement of renal function in potential living kidney donors (PLKD) is essential. The direct measurement of glomerular filtration rate (mGFR) has been considered the “gold standard.” The estimated GFR (eGFR) with 24-hour urinary creatinine clearance (CrCl) is frequently used because of its availability. We aim to evaluate the correlation and agreement of eGFR using serum-based creatinine formulas (Cockcroft–Gault, MDRD, and CKD-EPI) and the eGFR based on 24-hour urinary CrCl to evaluate kidney function in PLKD.

**Methods:**

We evaluated the kidney function in 799 PLKD using 24-hour urinary CrCl method and compared the correlation and agreement with the eGFR based on creatinine formulas (Cockcroft–Gault, MDRD, and CKD-EPI). We calculated the mean bias (difference), precision (SD of this difference), accuracy, and performed Bland–Altman plots.

**Results:**

A total of 799 PLKD were analyzed. The age of the PLKD ranged from 18 to 73 years. Weak to mild correlation was observed between 24-hour urinary CrCl and all formulas (ranged from 0.31 to 0.49). The three equations underestimated the GFR. Using the Bland–Altman graphic, we observed that the CKD-EPI was the least scattered and most precise; however, mean bias and the interval range (limits of agreement) of all formulas were too big to assume equivalence between 24-hour urinary CrCl method and eGFR based on creatinine. Results of mean bias were similar when comparing the three equations in patients with CrCl GFR <60. However, the accuracy of all formulas was better for the female group and the youngest individuals (≤40 years old).

**Conclusion:**

In this PLKD cohort, of all the three equations, the CKD-EPI was the least scattered and most precise. However, the correlation and the level of agreement between the three equations and 24-hour urinary CrCl were too low to assume the equivalence.

## 1. Introduction

The glomerular filtration rate (GFR) has been considered the best method to calculate global kidney function [1]. The inulin is the gold standard filtration marker for measured GFR (mGFR) because it is freely filtered from the glomeruli [2]. However, inulin filtration is an invasive method, and it is not cost-effective [3]. Hence, GFR estimating equations (eGFR) based on serum creatinine or cystatin C is the most common practice for use with patients [4].

The eGFR such as Cockcroft–Gault (CG), modification of diet in renal disease (MDRD), and chronic kidney disease epidemiology collaboration (CKD-EPI) are useful to calculate GFR and kidney function stratification [4–8]. Otherwise, serum creatinine depends on variables such as age, weight, muscle mass, demographic characteristics, and diet to compute eGFR. This dependence could overestimate or underestimate GFR [9]. In consequence, different studies have assessed the accuracy, bias, and agreement between eGFR by the Bland–Altman method and other concordance methods [10–12]. Some studies also evaluated different eGFR compared to 24 hours urine creatinine clearance (CrCl) [13, 14].

In kidney transplantation, the selection of potential living kidney donors (PLKD) must be a rigorous process where the eGFR is one of the main factors to determine PLKD eligibility [15]. GFR is routinely measured by standard 24 hours urinary creatinine clearance in PLKD [13]. Several methods of measured or estimated GFR try to predict PLKD kidney function pre and postnephrectomy [16] to avoid chronic kidney disease (CKD) risk in this population [17]. Nowadays, clinical practice guidelines are not clear about the ideal method to eGFR in PLKD [18].

In this study, we aimed at comparing the different eGFR with 24 hours urine CrCl in PLKD to determine their accuracy, bias, and agreement and how it may influence PLKD eligibility.

## 2. Methods

### 2.1. Study Population and Data Collection

This retrospective study was carried out with PLKD evaluated in Colombiana de Trasplantes from October 2008 to October 2020. Colombiana de Trasplantes is a transplant network in Colombia with 4 centers within the country that performs around 21% of the annual national kidney transplant activity. Inclusion criteria included patients older than 18 years old who were screened as potential donors. The potential donors were all the patients that submitted themselves to become living donors and were approved by our mental health team. The mental health team did a semistructured interview to assess the psychosocial status, disapproving the patients who had compromised legal requirements (competence, altruism, and complete information of risks), ethical principles (autonomy, justice, beneficence, and nonmaleficence), or psychological considerations (mature, stable, and understandable decision). Exclusion criteria for the study included 24 hours urine CrCl >180 mg/dl. Comorbidities were not considered exclusion criteria in the study but were a relevant factor for selecting effective donors. We studied all the PLKD, regardless of whether they became effective donors. We reviewed a total of 829 medical records. In case of implausibility or inconsistency, medical records and laboratory registers were reviewed for correction/verification. The final study population comprised 799 PLKD after applying the exclusion criteria. Data collection included demographic data, age, sex, race, weight, height, body mass index (BMI), and serum creatinine, and eGFR was estimated by the three equations (Cockcroft–Gault, MDRD, and CKD-EPI) and 24-hour urine creatinine clearance.

### 2.2. GFR Assessment

Based on the recommendations made by the kidney disease improving global outcomes (KDIGO) [16] and Organ Procurement and Transplantation Network (OPTN) guidelines for GFR assessment, our center requires 24-hour urinary CrCl to estimate GFR as part of evaluation of living donor candidates. Every potential donor is instructed to collect 24 hours urine in a container. The collected urine is analyzed by a certified lab and the GFR (based on 24 h urine creatinine clearance) is reported. The potential donor who has undercollections or overcollections was asked to collect the urine again. Serum creatinine was measured using an automated chemistry analyzer (expressed as mg/dl) and eGFR was calculated using the MDRD (ml/min/1.73 m^2^), Cockcroft–Gault (CG), and CKD-EPI (ml/min/1.73 m^2^) formulas. Definitions of estimation methods are shown in Table 1.

### 2.3. Kidney Donors

The policy of our center is that individuals who had a history of a primary renal or systemic disease known to affect kidney's function and GFR lower than 60 ml/min/1.73 m^2^ are not accepted for donation. Live kidney donors should have GFR (24 h urine creatinine clearance) equal to or higher than 90 ml/min/1.73 m^2^, donors with GFR between 60–89 ml/min/1.73 m^2^ will be assessed depends on age, demographics, and risk factors [16].

### 2.4. Statistical Analysis

Numerical data were expressed as medians or means and ranges or standard deviation according to distribution, and the categorical variables as numbers and percentages. To examine the correlation between 24 h urine CrCl and estimated GFR (MDRD, CKD-EPI, and CG) the Lin's concordance correlation coefficient was used (ranges from −1.0 to +1.0). The closer the coefficients are to +1.0 or −1.0, the greater the strength of the linear relationship is. Correlation degree was defined as weak 0.20–0.39, moderate 0.40–0.59, strong 0.60–0.79, and very strong 0.80–1.00.

To assess the agreement between the two measurement methods (24 h urine CrCl and estimated GFR with the three equations) we performed the Bland–Altman analysis. Bland and Altman quantified the difference between measurements using a graphical method. Furthermore, this method evaluates a bias between the mean differences, and it estimates an agreement interval, encompassing 95% of the differences observed in the second method when compared to the first method. Then, we drew a scatterplot in which the *X*-axis represented the average ((*K*1 + *K*2)/2) and the *Y*-axis represented the difference (*K*1 − *K*2) of two measurements. After the graph is drawn, the mean bias (mean of the *K*1 − *K*2) and its confidence limits (limits of agreement) are quantified [19].

Performance of each eGFR equation was assessed in terms of accuracy (bias), precision, and agreement. Bias was defined as the mean difference between measured (24 h urine CrCl) and estimated GFR (MDRD, CKD-EPI, and CG) and the standard deviation (SD) of this difference was defined as precision. Accuracy and precision were also evaluated separately for age groups (<40, >40), BMI (<25, >25), and gender.

These statistical limits were calculated by using the mean and the SD of the differences between the two measurements.

The difference between 24-hour urinary CrCl and eGFR was plotted against the mean of 24-hour urinary CrCl and eGFR. Bias and the 95% limits of agreement which were calculated as the mean difference ±1.96 times the precision were examined. Statistical Analysis Software R 4.0.3 was used for all analyses.

### 2.5. Ethics Considerations

This study was approved by the Institutional Research Committee, acting in concordance with national regulations, and International Regulations, such as the Declaration of Helsinki [20] and the Declaration of Istanbul [21]. None of the transplant donors were from a vulnerable population and all donors or next of kin provided written voluntary informed consent.

## 3. Results

A total of 799 PLKD were analyzed. Median age was 38.1 (range 18–73), 443 subjects were female, median of 24-hour urinary CrCl was 116 mg/dl (range 38.3–180 mg/dl), mean of eGFR was 110 ml/min/1.73 m^2^, 106 ml/min/1.73 m^2^, and 102 ml/min/1.73 m^2^ for CG, CKD-EPI, and MDRD equations, respectively. No significant differences were found in the mean of CKD-EPI or MDRD in Afro-American population. Demographics of the entire study cohort are described in Table 2.

### 3.1. CG Equations

After calculating the Lin's concordance correlation coefficient, weak to moderate correlation was observed between 24-hour urinary CrCl and all formulas with coefficients of *r*_MDRD_ = 0.31 (95% CI 0.2659–0.3681), *r*_CKD-EPI_ = 0.32 (95% CI 0.2744–0.3798), and *r*_CG_ = 0.49 (95% CI 0.4449–0.5469). Lin's concordance correlation coefficient did not show significant differences in terms of race (Black vs others) by the eGFR equations (CKD-EPI *r*_CKD-EPI_ = 0.32 and MDRD *r*_MDRD_ = 0.31).

After calculating the mean difference (bias) between measurements (24-hour urinary CrCl and each equation), these results showed that there was a positive difference for each equation (MDRD = 13.8, CKD-EPI = 9.8, and CG = 5.8), revealing that all of three equations tended to underestimate GFR.

CG gave the lowest bias of the three equations (bias of 5.8 compared to 13.8 and 9.8 for the MDRD and CKD-EPI, respectively), whereas CKD-EPI was the least scattered and most precise (SD 24.8; spread of data between lower and upper LoA (limits of agreement): 97.5 compared to 98.5 and 99.3 for the CG and MDRD, respectively).

Analyzing individuals by gender, BMI, and age groups separately, highest accuracy was found for the female and the youngest group for the three equations, whereas individuals with BMI >25 performance of CG and CKD-EPI was better compared to MDRD.  Table 3 describes the accuracy, precision, and agreement for the entire cohort and separated by subgroups.

The Afro-American race was analyzed by the eGFR equations that consider race (CKD-EPI and MDRD) in its estimation variables without any significant difference in bias (CKD-EPI bias 9.8 vs 9.6, MDRD bias 13.8 vs 14.29 without and with race) or Bland–Altman plots analysis.

**Figure 1:**
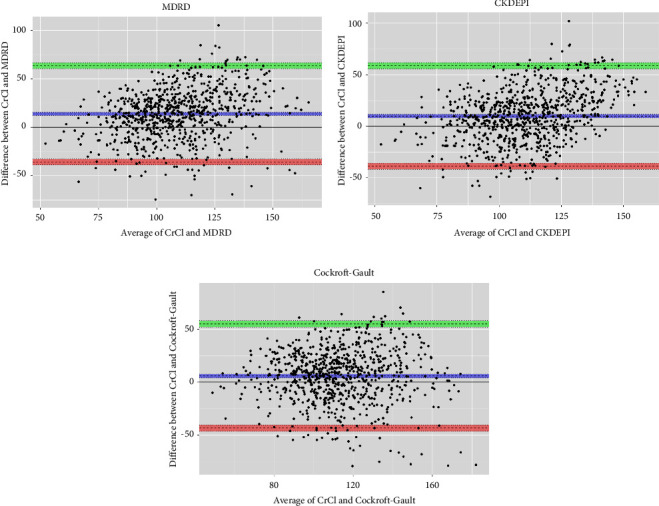
Comparison of the eGFR formulas with 24-hour urinary CrCl. (a) MDRD, (b) CKD-EPI, and (c) CG.

The Bland–Altman plots of the measured and estimated renal function with bias and 95% limits of agreement for each equation showed the smallest mean bias for the CG compared to the CKD-EPI, CG, and MDRD equations compared with 24-hour urinary CrCl (Figure 1).

## 4. Discussion

A crucial point in living kidney donation is the accurate evaluation of renal function in terms of ensuring donor safety and the best recipient outcomes. Many transplant centers evaluate the renal function of potential donors from CrCl. However, the clinical practice guidelines are not clear about the ideal method to estimate the GFR in PLKD.

The present study aimed to analyze the performance of the MDRD, CKD-EPI, and CG equations to estimate renal function among a cohort of PLKD by comparing estimates to GFR based on 24-hour urinary CrCl. Results of our study showed a weak to moderate correlation between 24-hour urinary CrCl and eGFR for all formulas. Moreover, all of them underestimated the GFR, while the CG provided a least biased estimate with the highest accuracy in the entire cohort. This was also true when the subjects were divided into subgroups (sex, BMI, and age), whereas the CKD-EPI was the least scattered and most precise. The interval range (limits of agreement) of all formulas was too big to assume equivalence between 24-hour urinary CrCl method and eGFR based on creatinine (Cockcroft–Gault, MDRD, and CKD-EPI).

Our study did not find significant differences in terms of race by the eGFR equations (CKD-EPI and MDRD). In a systematic review with 1064 studies, CKD-EPI had equal accuracy between white, black, or other races [22]. Kong et al. [23] published a cohort of 977 patients where CKD-EPI equation had better performance comparing two level-equation (white, black, or other races) than in 4 level-equation (hispanic, white, black, asian, and other). A cohort with 1988 patients reported that the Afro–American population had overestimated the eGFR-MDRD and eGFR-CKD-EPI when compared to white participants (*p*  <  0.001) [24].

Other studies looking at the performance of these equations have found inconsistent results. In a study from India on 173 kidney donors, Mahajan et al. [25] reported that in the prediction equations, the MDRD study equation is the most precise and accurate, whereas CG is the least biased. However, they conclude that error level exhibited by these equations makes them suboptimal for clinical use. In another study from Pakistan, Hafeez et al. [13] studied 207 potential live-related donors and they concluded that the CKD-EPI is closer to 24-hour urinary creatinine clearance in the calculation of eGFR. However, none of the eGFR formulas can be used in renal transplant donors because of their low accuracy. Lin et al. [26] evaluated 117 kidney donors and found out that the MDRD formula consistently underestimated GFR but was more accurate than the CG formula. Chaurasia et al. [27] evaluated the performance of GFR estimation equations among 51 healthy donors and found that both the CG and MDRD formulas underestimated GFR.

In our study, we found that the accuracy of all formulas was better for the female group and the highest accuracy was for the CG and the CKD-EPI formulas, being the CKD-EPI the most precise. Arreola–Guerra et al. [28] found that CKD-EPI is the most precise eGFR equation comparing with MDRD and the reference method Tc99DTPA in a Mexican population of 97 healthy patients. A study from Saudi Arabia with 31 patients compared MDRD and CKD-EPI equations with the inulin clearance in patients with CKD. They found that CKD-EPI equation was more precise than MDRD in this population [29]. Conversely, Hafeez et al. [13] and Michels et al. [30] found that the performance of the MDRD formula was better in males than females. A difference in race and renal function of patient populations can explain these discrepancies in different studies.

Hafeez et al. [13] assessed the influence of age on the performance of MDRD formula finding the highest accuracy in the youngest group (≤30 years old). With increasing age, the accuracy of MDRD formula declined because of greater degree of underestimation of GFR in older individuals. This resembles our study findings, where accuracy for all three formulas was higher in youngest individuals (≤40 years old) and the highest accuracy was found in the CG formula. In contrast to our study findings, Cirillo et al. [31] did not find any significant difference in the GFR estimation by the MDRD formula among different age groups (18–88 years). In our study, the performance of CKD-EPI was found to be better in overweight individuals. This is consistent with Lemoine et al. [32] that showed that the CKD-EPI equation is validated in the obese population up to a BMI range of 40 kg/m^2^.

Chaurasia et al. [27] from Nepal highlighted the importance of the 24-hour urinary creatinine clearance measurement reporting it as the most precise, highest accuracy, and highest Pearson correlation distinguish between MDRD and CG eGFR. In our study, 24-hour urinary clearance is the standard to compare the eGFR equations.

There are some weaknesses of this study. First, we studied a single Colombian population of PLKD and it is unlikely that a single equation will work well in all populations. Second, there were few participants older than 70 years of age or racial minorities and the study population is not representative of the general population. Furthermore, this is a cohort of PLKD that were generally healthy, and these findings could not be generalized to the general population. Finally, comparison between measurements do not overcome limitations of serum creatinine as an endogenous filtration marker. Strengths included that to the best of our knowledge, ours is the first study that validate these equations in a Colombian cohort of PLKD.

## 5. Conclusion

We conclude that **i**n this PLKD cohort, of all the three equations, CKD-EPI was the least scattered and most precise. However, the three equations underestimated the GFR. These may misclassify the healthy individuals into chronic kidney disease population. The correlation and the level of agreement between the three equations and 24-hour urinary CrCl were too low to assume equivalence. Therefore, the use of the three equations in the evaluation of renal function among living kidney donor candidates must be performed with caution. The accuracy of all formulas was better for the female group and the youngest individuals (≤40 years old).

## Figures and Tables

**Table 1:** Definition of estimation methods.

Estimation method	Equation
MDRD	175 × creatinine [mg/dl]^−1.154^ × age [years]^−0.203^ × 0.742 [if female]
CKD-EPI	141 × min {creatinine/*k*, 1}^*α*^ × max {creatinine/*k*, 1}^−1.209^ × 0.993^age [years]^ × 1.018 [if female] _ 1.159 [if black] where *k* is 0.7 for females and 0.9 for males, *α* is −0.329 for females and −0.411 for males
CG	((140 − age (years)) × body weight (kg))/creatinine [*μ*mol/l] × 0.85 [if female]

Equations for estimating renal function based on serum creatinine. MDRD: modification of diet in renal disease, CKD-EPI: chronic kidney disease epidemiology collaboration; CG: Cockcroft–Gault.

**Table 2:** Demographics of the entire study cohort.

Characteristics of the study population	Total (*N* = 799)
Sex, *n* (%)	
Male	356 (44.6)
Female	443 (55.4)
Age, years, mean (SD)	38.1 (11.6)
Age groups, *n* (%)	
18–40	454 (56.8)
40–60	320 (40.1)
60–70	23 (2.9)
>70	2 (0.3)
Race, *n* (%)	
Hispanic	688 (86.1)
Caucasian	87 (10.9)
Afro-American	16 (2)
Others	5 (0.6)
Unknown	3 (0.4)
Weight, kg, mean (SD)	66.9 (11.7)
Height, cm, mean (SD)	164 (8.77)
BMI, kg/m^2^, mean (SD)	25.0 (3.53)
BMI groups, *n* (%)	
<25	419 (52.4)
>25	380 (47.6)
24-hour urinary CrCl (SD)	116 (25.5)
eGFR CG (SD), ml/min/1.73 m^2^	110 (25.3)
eGFR CKD-EPI mean (SD), ml/min/1.73 m^2^	106 (17.8)
eGFR CKD-EPI mean (SD), ml/min/1.73 m^2^, Afro-American	106 (18)
eGFR MDRD mean (SD), ml/min/1.73 m^2^	102 (19.5)
eGFR MDRD mean (SD), ml/min/1.73 m^2^, Afro-American	101 (19)

Note: SD: standard deviation; kg: kilograms; cm: centimeters; m: meter; BMI: body mass index; eGFR: estimated glomerular filtration rate; ml: milliliters; min: minute.

**Table 3:** Accuracy, precision, and agreement comparing the MDRD, CKD-EPI, and CG equations for the entire cohort and separated by subgroups.

Estimation method	Accuracy and precision	Agreement	
	Bias (SD bias)	Upper limit of agreement (ULoA) (95% CI)	Lower limit of agreement (LLoA) (95% CI)
MDRD	13.8 (25.3)	63.5 (60.5 to 66.5)	−35.8 (−38.8 to −32.8)
Male	19.5 (27.0)	72.4 (67.6 to 77.2)	−33.3 (−38.1 to −28.5)
Female	9.2 (22.9)	54.2 (50.6 to 57.9)	−35.7 (−39.4 to −32.0)
BMI >25	20.3 (26.2)	71.6 (67.1 to 76.1)	−31.0 (−35.5 to −26.5)
BMI <25	7.9 (23.0)	53.1 (49.3 to 56.9)	−37.1 (−40.9 to −33.4)
Age <40	11.9 (26.2)	63.3 (59.1 to 67.4)	−39.4 (−43.5 to −35.2)
Age >40	16.3 (23.9)	63.3 (58.9 to 67.6)	−30.6 (−34.9 to −26.2)
CKD-EPI	9.8 (24.8)	58.6 (55.7 to 61.6)	−38.8 (−41.8 to −35.9)
Male	17.7 (25.4)	67.7 (63.1 to 72.2)	−32.2 (−36.7 to −27.6)
Female	3.5 (22.5)	47.6 (44.1- to 51.2)	−40.5 (−44.1 to −36.9)
BMI >25	16.6 (25.3)	66.3 (61.9 to 70.7)	−33.1 (−37.4 to −28.7)
BMI <25	3.7 (22.7)	48.4 (44.7 to 52.2)	−40.8 (−44.6 to −37.1)
Age <40	6.7 (25.4)	56.6 (52.6 to 60.6)	−43.0 (−47.0 to −39.0)
Age >40	13.9 (23.5)	60.1 (55.9 to 64.4)	−32.2 (−36.5 to −27.9)
CG	5.8 (25.1)	55.1 (52.1 to 58.0)	−43.4 (−46.3 to −40.4)
Male	8.7 (24.9)	57.7 (53.2 to 62.2)	−40.2 (−44.6 to −35.7)
Female	3.5 (25.0)	52.5 (48.5 to 56.5)	−45.5 (−49.5 to −41.5)
BMI >25	0.2 (27.3)	53.8 (49.0 to 58.5)	−53.2 (−58.0 to −48.5)
BMI <25	10.9 (21.7)	53.6 (50.0 to 57.2)	−31.7 (−35.3 to −28.2)
Age <40	1.6 (25.9)	52.5 (48.4 to 56.6)	−49.3 (−53.4 to −45.2)
Age >40	11.4 (22.8)	56.1 (52.0 to 60.2)	−33.2 (−37.4 to −29.1)

Note: SD: standard deviation; ULoA: upper limit of agreement; LLoA: lower limit of agreement; CI: confidence interval; MDRD: modification of diet in renal disease; CKD-EPI: chronic kidney disease epidemiology collaboration; CG: Cockcroft–Gault.

## Data Availability

The data supporting this study are available on request from the corresponding author.

## Abbreviations

GFR: Glomerular filtration rate
mGFR: Measured glomerular filtration rate
eGFR: Glomerular filtration rate estimating equations
CG: Cockcroft–Gault
CKD-EPI: Chronic kidney disease epidemiology
MDRD: Modification of diet in renal disease
CrCl: Creatinine clearance
PLKD: Potential living kidney donors
BMI: Body mass index
KDIGO: Kidney disease improving global outcomes
OPTN: Organ Procurement and Transplantation Network
SD: Standard deviation
LoA: Limits of agreement
ULoA: Upper limit of agreement
LLoA: Lower limit of agreement.
